# Correlation between Neutrophil-to-Lymphocyte Ratio and Cerebral Edema in Children with Severe Diabetic Ketoacidosis

**DOI:** 10.3390/biomedicines11112976

**Published:** 2023-11-05

**Authors:** Alexandra-Cristina Scutca, Delia-Maria Nicoară, Niculina Mang, Iulius Jugănaru, Giorgiana-Flavia Brad, Otilia Mărginean

**Affiliations:** 1 Department XI Pediatrics, Discipline I Pediatrics, ‘Victor Babeş’ University of Medicine and Pharmacy of Timisoara, 300041 Timisoara, Romania; scutca.alexandra@umft.ro (A.-C.S.); nina.mang@umft.ro (N.M.); juganaru.iulius@umft.ro (I.J.); brad.giorgiana@umft.ro (G.-F.B.); marginean.otilia@umft.ro (O.M.); 2 Department of Pediatrics I, Children’s Emergency Hospital “Louis Turcanu”, 300011 Timisoara, Romania; 3 Research Center for Disturbances of Growth and Development in Children BELIVE, ‘Victor Babeş’ University of Medicine and Pharmacy of Timisoara, 300041 Timisoara, Romania

**Keywords:** severe DKA, cerebral edema, NLR, inflammation

## Abstract

Diabetic ketoacidosis (DKA), a common onset modality of type 1 diabetes mellitus (T1DM), can lead, in rare instances, to the development of cerebral edema, which is the leading cause of mortality in T1DM. Aside from the identification of several demographic and clinical risk factors for cerebral edema, attention has also been drawn to the possible link between systemic inflammation and neuroinflammation. This single-center retrospective study of 98 children with severe DKA aimed to investigate the possible relationship between neutrophil-to-lymphocyte ratio NLR) levels and the presence of cerebral edema. Patients were classified into three groups: alert (*n* = 28), subclinical cerebral edema (*n* = 59), and overt cerebral edema (*n* = 11). Lower blood pH and elevated NLR and blood urea were correlated with the presence of cerebral edema (*p* < 0.001). After a multivariable risk adjustment for possible confounding factors, such as age, pH, corrected sodium, and BUN, the NLR remained positively associated with cerebral edema (*p* = 0.045). As such, NLR may be an additional instrument to help practitioners target patients with a higher risk of severe cerebral edema. These patients would benefit from more rigorous neurologic surveillance, enabling the prompt identification of early signs of cerebral edema.

## 1. Introduction

Diabetic ketoacidosis (DKA), a common form of onset in type 1 diabetes mellitus (T1DM), can lead in severe cases to devastating consequences due to the risk of symptomatic cerebral edema [1]. This rare complication occurs in approximately 1% of DKA cases [2,3,4], usually during the first 12 h of treatment [5,6]. However, it may become apparent even before therapy is started [7,8,9], and prompt recognition and management are vital for a favorable outcome [2]. Researchers have focused on identifying the exact profile of children that are prone to this neurologic complication, and have proposed mostly demographic and biochemical risk factors [10]. Previous studies have acknowledged the role of inflammation in both acute [11] and chronic [12] diabetic complications. During the last decade, several studies have reported an increased inflammatory burden in cerebral edema [13], and have investigated a potential connection between systemic inflammation and neuroinflammation in the context of DKA [14,15,16,17,18,19]. Furthermore, in severe DKA, vasoactive substances released by active systemic neutrophils have been noted to potentially disrupt the blood–brain barrier (BBB) [20]. DKA, recognized as a state of systemic inflammation [21,22], escalates the risk of cerebral edema [2]. The neutrophil-to-lymphocyte ratio (NLR), an established inflammatory marker associated with diabetes mellitus, stands as a pivotal indicator of inflammation in both chronic and acute diabetic complications [23,24,25]. Our study focused on characterizing children with severe DKA, with particular focus on the relationship between NLR and the presence of cerebral edema.

## 2. Materials and Methods

### 2.1. Study Design and Protocol

This hospital-based retrospective cross-sectional study was performed in one large reference center for pediatric diabetes in Romania. We reviewed 104 patients diagnosed with severe diabetic ketoacidosis (DKA) in the Pediatric Emergency Hospital “Louis Turcanu” in Timisoara, Romania, between 1 January 2015 and 30 June 2023. Our study protocol, performed in accordance with Good Clinical Practice (Declaration of Helsinki from 1975, revised in 2013), was approved by the Local Ethics Committee; given the retrospective nature of the study, informed consent was waived in accordance with ethical regulations. Inclusion criteria consisted of (1) age below 18 years, (2) diagnosis of new-onset/established T1DM according to the American Diabetes Association (ADA) criteria of 2021, and (3) severe DKA. Exclusion criteria were as follows: (1) patients with incomplete medical documentation, and (2) children with preexisting hematologic or neurological abnormality. Severe DKA was diagnosed as plasma glucose value > 11 mmol/L, and an arterial pH value < 7.10 or serum bicarbonate < 5 mmol/L at the time of admission [26]. We assessed the presence and severity of DKA-related mental status alteration by evaluating patients’ Glasgow Coma Scale (GCS) scores. Patients with overt cerebral edema were considered to be those with GCS score < 14 and an alteration in neurologic status which required hypertonic solution or intubation [1]. Children with Glasgow Coma Scale (GCS) scores of 13 or 14 that did not fulfill the criteria for overt cerebral edema were assigned to the subclinical cerebral edema group.

### 2.2. Clinical and Laboratory Data

Patient characteristics were collected, including age, gender, weight status (underweight, normal weight, overweight), physical examination upon admission (including the evaluation of impaired consciousness using GCS score), duration of symptoms, and presence of family history of DM. Underweight was considered as body mass index (BMI) < 5th percentile, while overweight was >85th percentile according to online Centers for Disease Control and Prevention (CDC) BMI charts [27]. Blood pressure percentiles were calculated according to age and height-specific charts. Laboratory parameters upon admission included complete blood count (CBC), using a Sysmex XN-550 automated hematology analyzer (Sysmex Corporation, Kobe, Japan) and biochemistry tests. The latter, including C-reactive protein (CRP), fasting glucose level, blood urea nitrogen (BUN), creatinine, and High-density lipoprotein cholesterol (HDL-c), were evaluated using an automatic analyzer (Hitachi 747, Hitachi, Tokyo, Japan). Glycated Hemoglobin (HbA1c) was evaluated using high-performance liquid chromatography (Cobas E 411—Roche, Tokyo, Japan). Peptide C and insulinemia were assessed using an automated chemiluminescent assay (Cobas E 411—Roche, Tokyo, Japan). NLR score was calculated based on peripheral blood cell counts, as neutrophil count divided by lymphocyte count [28]. The formula used for effective serum osmolality was 2 × (Na + K) + glucose/18 [29]. Sodium was adjusted for hyperglycemia, and calculated as Na + 1.6 × (glucose − 100)/100 [30].

### 2.3. Statistical Analysis

Patients were divided into three study groups, according to the presence and severity of cerebral edema, and characterized using descriptive statistics [percentage, median, range of quarters]. The analytical Kolmogorov–Smirnov test was used to assess the extent to which the data followed a normal distribution. Numerical variables displayed non-normal distribution, and were plotted as median (25th and 75th IQR) and compared across groups using the Kruskal–Wallis test. Categorical variables were plotted as numerical and percentile values, and compared across groups using the Chi-squared test. The Spearman rank correlation coefficient (*r*) was employed to assess the relationships between different clinical parameters and the severity of altered mental status. Furthermore, multiple linear regression analysis was used to explore whether NLR was independently associated with the presence of altered mental status. Finally, a receiver operating characteristic (ROC) curve was used to further characterize the diagnostic value of the NLR in identifying patients with altered mental status, using ROC curves and the calculation of proper cut-off values (Youden’s index, calculated as sensitivity + specificity − 1). Statistical analysis was performed using Statistical Package for Social Sciences software (SPSS v28.0.1.1., IBM Corporation, Armonk, NY, USA ). A two-tailed *p*-value < 0.05 was considered statistically significant.

## 3. Results

### 3.1. Patient Characteristics Stratified by the Severity of Neurological Impairment

#### 3.1.1. General Description of the Study Lot

The study group included a total of 98 children with severe DKA, from which 86 had new onset T1DM and 12 were previously diagnosed cases. Children were dichotomized into alert (28 patients), subclinical cerebral edema (59 patients), and overt cerebral edema (11 patients). Demographic and clinical data of these children are illustrated in Table 1. The median age of the entire study lot was 9.7 [interquartile range (IQR): 11–15] years old, with no significant differences across study groups, although patients with altered mental status tended to be smaller. There was also a trend toward female prevalence with the aggravation of neurologic impairment, without reaching statistical significance (*p* = 0.186). In addition, blood pressure increased significantly with the aggravation of neurologic impairment. As expected, children with cerebral edema had a longer duration of PICU stay and time to correction of acidosis (*p* < 0.001).

#### 3.1.2. Laboratory Parameters upon Admission

Differences in blood gas analysis and metabolic panels across study groups are summarized in Table 2. Patients with signs of cerebral edema had more pronounced metabolic acidosis (*p* < 0.001), more elevated corrected sodium levels (*p* = 0.019), osmolality (0.005), and blood urea nitrogen (*p* = 0.002). The study groups had similar mean glycemia, HbA1c, insulinemia, and basal C-peptide, although a trend toward a gradual decrease in plasma insulin and basal C-peptide levels could be noted with the aggravation of neurologic impairment.

In addition, there was significant heterogeneity between groups in terms of CBC parameters (Table 3), most notably regarding neutrophils, monocytes, and immature granulocytes (*p* = 0.001, *p* <0.001, and *p* = 0.012, respectively).

**Figure 1:**
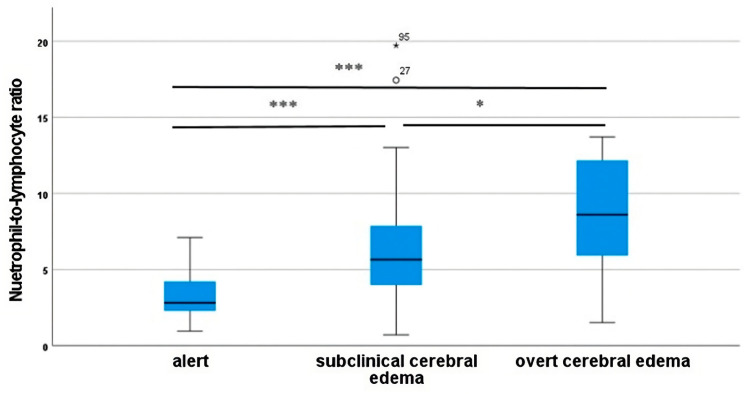
NLR scores across study groups using univariate analysis with post hoc procedure; *** *p* < 0.05, * *p* > 0.05. ★ far out outliers; ◦ mild outliers.

Median NLR scores increased gradually from those without neurologic impairment (2.82) to those with subclinical and overt cerebral edema (5.66 and 8.60, respectively) (Table 3, Figure 1). Subsequently, a post hoc analysis revealed significant differences in median NLR scores between those alert and those with subclinical (*p* < 0.001) and overt cerebral edema (*p* < 0.001). However, median NLR scores were similar between those with subclinical and those with overt cerebral edema (*p* = 0.292).

### 3.2. Correlation and Association Analysis between Different Clinical Parameters and Cerebral Edema

Lower blood pH and elevated NLR and blood urea were correlated with the presence of cerebral edema (*p* < 0.001); also, we noted a weak correlation between the presence and severity of cerebral edema and elevated levels of corrected sodium and associated co-infection. A regression analysis was further employed to investigate the independence of NLR in predicting the presence of cerebral edema. As depicted in Table 4, after multivariable risk adjustment for possible confounding factors, such as age, pH, corrected sodium, and BUN, the NLR remained positively associated with cerebral edema (*p* = 0.045).

### 3.3. Diagnostic Performance of NLR

**Figure 2:**
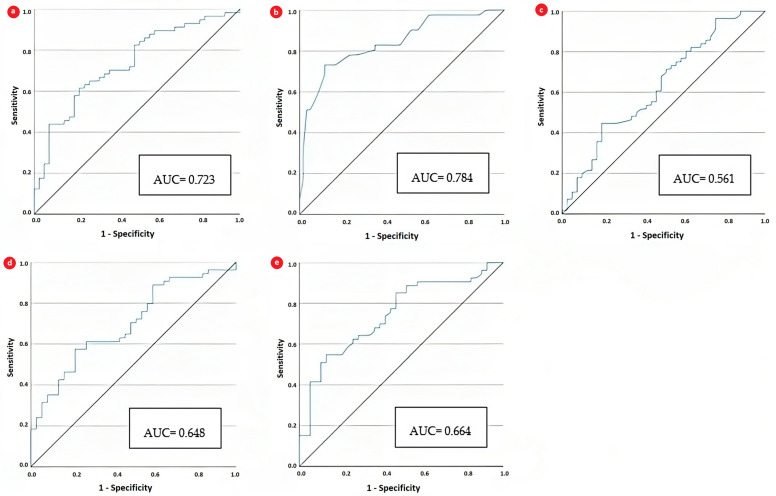
Illustration of the area under the curve of (**a**) NLR, (**b**) blood pH, (**c**) glycemia, (**d**) osmolality, and (**e**) BUN for predicting altered mental status.

We plotted receiver operating characteristic (ROC) curves to assess the discriminatory ability of NLR and compare it to the ROC curves of pH, glycaemia, corrected sodium, osmolality, and BUN in distinguishing cerebral edema across the entire study lot (Figure 2).

Blood pH had superior diagnostic discrimination, with the largest area under the curve (AUC) at 0.784 [AUC: 0.784, (0.682–0.886), sensitivity 77.4%, specificity 55.2%]. NLR also displayed acceptable discriminatory power, with an AUC of 0.723. The threshold values according to Youden’s index for pH and NLR were 6.96 and 4.05, respectively (Table 5).

## 4. Discussion

In the course of our investigation, we observed a notable association wherein elevated NLR values were consistently identified in the pediatric subgroup with cerebral edema. This finding highlights the potential significance of NLR as an additional marker in the clinical evaluation of cerebral edema.

It is widely acknowledged that neurologic complications are the leading cause of mortality in children with DKA, with cerebral edema accounting for 40–90% of pediatric diabetes-related deaths [31]. Given the high fatality rate, efforts have been made to identify the underlying mechanisms leading to cerebral edema and the potential risk factors. However, the interpretation of research findings remains a matter of debate, even after more than 70 years after the initial description [32]. The three main types of cerebral edema that may complicate childhood DKA are cytotoxic [5,33,34,35,36,37,38,39,40], osmotic [38,41,42,43,44], and vasogenic edema [34,36,44,45]. Several studies suggest that cytotoxic edema is the consequence of cerebral hypoperfusion, secondary to hypocapnia and severe dehydration [39,46]; in addition, single photon emission tomographic studies have demonstrated a baseline cerebral hypoperfusion in children with DKA, which, in the presence of hypocapnia and dehydration, can lead to ischemic injury [33,47]. Although osmotic injury from aggressive fluid resuscitation or insulin administration in the first hour of therapy has historically been considered a major factor in the development of DKA-related cerebral edema [42,48,49,50], recent studies have failed to confirm this hypothesis [5,40]. Vasoactive substances released in response to hyperglycemia and hyperketonemia cause a disruption of the blood–brain barrier, and thus vasogenic edema [51,52]. The latter is the main finding in children with subclinical cerebral edema, as evidenced by increased water diffusion in diffusion-weighted imaging studies [53,54]. Some studies propose a sequential order of events [55,56,57], while others consider there to be a complex interplay and overlap between the three types of edema [58]. In addition, research has focused on identifying specific risk factors responsible for the development of DKA-related cerebral edema, such as new-onset DKA [7,39,59,60], young age [61], a longer duration of symptoms [59,62], hypocapnia [5,37,62,63,64,65,66], hyperosmolality [67,68,69], elevated blood urea nitrogen [5,37,65,70,71,72,73], dehydration [62,64], and severe acidosis [4,37,48,59,65,66,72,74,75,76,77,78,79].

Similar to previous reports [65,66], in the current study low blood pH and elevated blood urea nitrogen were associated with the development of cerebral edema, as assessed by regression analysis. However, we extended the characterization of blood investigations more frequently associated with the presence of DKA-related cerebral edema by analyzing the CBC parameters of our study groups. As such, we noticed an increase in NLR values with the aggravation of neurologic impairment. Regarding the importance of peripheral inflammation in neurologic complications, Ferro et al. previously described a link between elevated NLR values and the onset of cerebral edema after reperfusion therapy in adult patients with ischemic stroke [13]. According to Close et al., elevated levels of IL-6, IL-8(KC), and MCP-1 are suggestive for the presence of a systemic inflammatory response during DKA with the potential to induce cerebrovascular endothelial dysfunction and leukocyte activation [80]. Another study on animals, by Lin et al., concluded that hyperglycemia might trigger the migration of neutrophils into the brain tissue, in the context of transient cerebral ischemia [81]. Human studies have also described a possible link between peripheral and cerebrovascular inflammation, contributing to vasogenic lesions [20,82,83,84]. Woo et al. suggested that active polymorphonuclears can cause BBB disruption, and thus vasogenic edema, in the context of DKA [20]. Omatsu et al. suggested that chemokines such as CXCL1/CXCL8 (GROalpha/IL-8) mediate the adhesion of leukocytes to the vascular endothelium, potentially leading to DKA-related cerebrovascular complications [82]. To the best of our knowledge, this is the first article to address the relationship between NLR and the presence of cerebral edema in children with severe DKA.

Regarding the demographic characteristics of the subgroups, although there were no significant statistical differences, children that developed overt cerebral edema were more likely to be smaller, with a median age of 9 years (IQR: 2.4, 12.1), of female gender (73%), and with a longer duration of symptoms. A similar age group and female prevalence were described in children with complicated DKA [61]. In total, 11.2% of the entire study lot presented with a clinical picture of symptomatic cerebral edema. Abbas et al. described a higher incidence rate of 43.2% cases with symptomatic cerebral edema. The difference could be related to the applied inclusion criteria, as only complicated severe DKA children admitted to the Pediatric Intensive Care Unit were taken into account [61].

In terms of clinical parameters, one in two children with overt cerebral edema from our study lot had coinfections (mainly respiratory, cutaneous, and urinary tract infections, and only one case with COVID). Poovazhagi et al. described a higher prevalence of cerebral edema in children with coinfection, as opposed to those without infections [85]. Although infections are well known to elevate NLR, we included patients with coinfections, as they represented one in five children and offered a more realistic and complete picture of patients with severe DKA. Surprisingly, NLR values did not display significant statistical differences when comparing children with and without infections (*p* = 0.774). This could partially be explained by the difference in the number of patients with and without infections (76 and 22, respectively). However, neutrophilia in these cases is also caused by other factors besides infection, such as the release of pro-inflammatory cytokines, elevated endogenous cortisol, and catecholamines [86,87]. Due to the retrospective nature of the study, we were not able to account for these possible confounders, as they were not part of the investigations collected during admission.

Another clinical characteristic of children with overt cerebral edema from our study lot was higher systolic and diastolic blood pressure. This is consistent with previous results that described hypertension as part of the clinical picture of children with DKA-related cerebral edema [88,89]. None of the children with overt cerebral edema from our lot died, as opposed to the mortality of 21–28% of cases cited by the literature [64,72].

As opposed to overt cerebral edema, which has a more precise definition [1], subclinical cerebral edema remains a matter of debate [32]. Children from our study lot with Glasgow Coma Scale (GCS) scores of 13 or 14 who did not fulfill the criteria for overt cerebral edema were assigned to the subclinical cerebral edema group. Concerning this issue, a clinical diagnosis based on minimal symptoms (obtundation, lethargy), without imagistic confirmation, may provoke controversy [90] as it is difficult to interpret the clinical picture of small children objectively. Moreover, these symptoms could be similar to those of severe dehydration [91]. Still, some particularities regarding their profile deserve mentioning. A slight tendency towards higher age, less female predominance, lower BP and HR values, and higher blood pH, glycemia, basal C-peptide, and NLR was noted, as opposed to the overt cerebral edema group, but without reaching the values of those from the alert group. This intermediary demographic and clinical profile is consistent with the intermediary neurologic profile, which situated them between the alert and the overt cerebral edema groups. Also, several studies demonstrate imagistic abnormalities in children with DKA and mild mental status disorders [5,33,37,54,65,66,78,92,93].

In summary, the presence of more elevated NLR values in children with DKA-related cerebral edema underscores its potential auxiliary role in different aspects of DKA management. First, in risk assessment, higher NLR in such children could indicate a subgroup of patients at higher risk of developing cerebral edema and in need of more vigilant monitoring. Also, this observation could spark further research into mechanisms linking elevated NLR and cerebral edema in T1DM DKA; understanding these mechanisms could lead to targeted therapies, aiming to improve outcomes and reduce the risk of severe complications.

There are some limitations to this study that deserve mentioning. First, it is a single-center, retrospective study, and therefore subject to inherent selection bias. In addition, we did not exclude cases with acute infections, in order to offer a more practical value to the study by reflecting on real-life situations, in which a significant number of patients with DKA have co-infections. Furthermore, cerebral edema was assessed solely based on clinical evaluation, lacking access to advanced imaging techniques. This limitation introduced inherent uncertainty, particularly concerning the identification of subclinical edema. However, cerebral edema is considered primarily a clinical diagnosis [32,64,65] and can be present in some cases in the absence of evident imagistic findings [62]. In addition, we could not include pCO2 in our analysis because baseline pCO2 values were not accessible for the entire cohort. Lastly, the relatively low sample size might overestimate the importance of the results. Therefore, prospective multi-center studies with imagistic confirmation of DKA-related cerebral edema are to be considered.

## 5. Conclusions

In summary, NLR might be of additional help to target patients that have a higher risk of severe cerebral edema and need more rigorous neurologic surveillance in order to identify initial warning signs and start specific treatment promptly.

## Figures and Tables

**Table 1:** Demographic and clinical characteristics of patients.

Variables	Alert (*n* = 28)	Subclinical Cerebral Edema (*n* = 59)	Overt Cerebral Edema (*n* = 11)	*p* Value
Age (years)	10.2 (4.8, 14)	9.60 (5.03, 13.01)	9 (2.4, 12.1)	0.637
Males % (*n*)	64.3 (18)	42.4 (25)	27.3 (3)	0.186
Duration of symptoms (days)	14 (10, 24.6)	10 (7, 16)	14 (6, 30)	**0.099**
New onset DKA	89.3 (25)	84.7 (50)	91.6 (11)	0.351
Overweight % (*n*)	10.7 (3)	10.2 (6)	18.2 (2)	0.597
Underweight % (*n*)	28.5 (8)	20.3 (12)	9.1 (1)	0.616
Co-infection % (*n*)	21.4 (6)	16.9 (10)	54.5 (6)	**0.022**
Vital signs at presentation,				
SBPp (mmHg)	92 (64.5, 97)	99 (95, 100)	100 (98, 100)	**0.002**
DBPp (mmHg)	86 (62.5, 97)	95 (76, 99)	96 (90, 100)	0.076
HR (beats/min)	111.5 (97.6, 128.9)	130 (117, 142)	131 (120, 142)	**0.002**
RR (respiration/min)	30 (23.2, 35)	34 (27.5, 39)	31 (30, 33)	0.258
Insulin before admission % (*n*)	10.7 (3)	15.3 (9)	27.3 (3)	0.308
Duration of PICU stay (days)	1 (1, 2)	2 (1, 2.5)	4 (3, 4)	**<0.001**
Time to correction of acidosis (hours)	9 (7, 12.7)	21 (16, 25)	28 (26, 38)	**<0.001**

Data are expressed as mean ± standard deviation, median (interquartile range, IQR), or percentage (n, %). DKA, diabetic ketoacidosis; SBPp, percentile of systolic blood pressure; DBPp, percentile of diastolic blood pressure; HR, heart rate; RR, respiratory rate; PICU, pediatric intensive care unit. Statistically significant differences, with a probability value of *p* < 0.05, are represented in bold.

**Table 2:** Blood gas analysis and metabolic panels upon admission.

Variables	Alert (*n* = 28)	Subclinical Cerebral Edema (*n* = 59)	Overt Cerebral Edema (*n* = 11)	*p*-Value
Venous pH	7.07 (6.97, 7.08)	6.97 (6.98, 7.01)	6.84 (6.80, 6.93)	**<0.001**
Glycemia (mg/dL)	445 (383, 532)	478 (381, 529)	557 (464, 590)	0.087
Base excess (mmol/L)	−23.5 (−26, −22)	−26.3 (−28.2, −25)	−29.8 (−30.9, −27.9)	**<0.001**
Lactate (mmol/L)	1.78 (1.34, 2.80)	2.30 (1.49, 3.02)	1.93 (1.13, 2.02)	0.170
Corrected sodium (mmol/L)	144.7 (142.2, 147.2)	145.4 (143.5, 147.8)	148.8 (146.5, 151,8)	**0.019**
Osmolality (mosmol/L)	306.7 (301.7, 310.6)	309.9 (303.4, 317.8)	320.8 (311.9, 329.5)	**0.005**
Creatinine (umol/L)	53 (44, 66)	56.5 (39.7, 73.2)	55 (28, 97)	0.926
HbA1c (%)	11.55 (12.62, 14.92)	11.40 (10.15, 12.68)	11.31 (10.25, 12.45)	0.563
Insulinemia (uIU/mL)	3.82 (2.39, 12.95)	2.19 (1.14, 11.07)	1.90 (1.67, 15.7)	0.156
Basal C-peptide (nmol/L)	0.402 (0.182, 0.721)	0.338 (0.218, 0.472)	0.265 (0.183, 0.508)	0.605
CRP (mg/L)	1.18 (0.39, 7.87)	2.07 (0.82, 7.09)	12.4 (1.78, 27.3)	0.078
BUN (mmol/L)	22.4 (15.5, 26.1)	24.7 (18.6, 35.5)	35.1 (29.5, 50.9)	**0.002**

Data are expressed as mean ± standard deviation, median (interquartile range, IQR), or percentage (n, %). HbA1c, glycated hemoglobin A1c; CRP, C-reactive protein, BUN, blood urea nitrogen. Statistically significant differences, with a probability value of *p* < 0.05, are represented in bold.

**Table 3:** Hematological parameters upon admission.

Variables	Alert (*n* = 28)	Subclinical Cerebral Edema (*n* = 59)	Overt Cerebral Edema (*n* = 11)	*p*-Value
WBC (×10^3^/mm^3^)	14.4 (10.7, 16.6)	18.8 (13.8 25.5)	18.5 (8.64, 24.5)	**0.008**
Neutrophils (×10^3^/mm^3^)	9.34 (6.29, 12.1)	14.7 (9.80, 20.7)	14.7 (9.22, 19.6)	**0.001**
Lymphocytes (×10^3^/mm^3^)	2.95 (2.36, 4.03)	2.59 (1.96, 3.91)	1.71 (0.97, 2.91)	**0.042**
Monocytes (×10^3^/mm^3^)	1.21 (0.85, 1.60)	1.69 (1.14, 2.47)	2.37 (1.85, 2.78)	**<0.001**
Basophiles (×10^3^/mm^3^)	0.04 (0.03, 0.05)	0.04 (0.03, 0.07)	0.03 (0.02, 0.05)	0.266
Eosinophiles (×10^3^/mm^3^)	0.02 (0, 0.12)	0.01 (0, 0.03)	0 (0, 0.01)	**0.026**
Immature granulocytes % (×10^3^/mm^3^)	0.07 (0.03, 0.13)	0.15 (0.06, 0.37)	0.21 (0.09, 0.42)	**0.012**
Platelets (×10^3^/mm^3^)	328 (294, 402)	342 (275, 440)	327 (275, 400)	0.992
NLR	2.82 (2.28, 4.23)	5.66 (3.95, 7.88)	8.60 (4.73, 12.17)	**<0.001**

Data are expressed as median (interquartile range, IQR). WBC, white blood cells, NLR, neutrophil-to-lymphocyte ratio. Statistically significant differences, with a probability value of *p* < 0.05, are represented in bold.

**Table 4:** Correlation of different clinical parameters with the presence and severity of altered mental status.

	Univariate Model (Groups 1 to 3)		Multivariate Model (Group 1 vs. 2+) r^2^ = 0.265	
Variables	Correlation Coefficient	*p*	B (95%CI)	*p*
Age *^r^*	−0.077	0.449	−0.018 (−0.038, 0.003)	0.088
Gender ^χ2^	0.182	0.073		
Coinfection ^χ2^	0.186	0.071		
pH *^r^*	−0.528	**<0.001**	−1.326 (−2.325, −0.327)	**0.010**
Glycemia *^r^*	0.173	0.090		
HbA1c *^r^*	0.092	0.288		
Corrected sodium * *^r^*	0.248	**0.016**	−0.003 (−0.025, 0.019)	0.815
BUN *^r^*	0.346	**<0.001**	0.007 (0.001, 0.013)	**0.042**
NLR *^r^*	0.404	**<0.001**	0.030 (0.001, 0.060)	**0.045**

Abbreviations: NLR, neutrophil/lymphocyte ratio; BUN, blood urea nitrogen; *^r^* Spearman’s Rho. coefficient; ^χ2^ chi-square test for independence. Group 1—alert; group 2—subclinical cerebral edema; group 3—overt cerebral edema; * corrected for blood glucose. Statistically significant differences, with a probability value of *p* < 0.05, are represented in bold.

**Table 5:** Comparison of diverse clinical parameters in discriminating altered mental status.

	AUC	SE	95%CI	Sensitivity	Specificity	Cut-Off	*p*-Value
NLR	0.723	0.051	0.619–0.827	0.716	0.538	4.05	**<0.001**
pH	0.784	0.052	0.682–0.886	0.774	0.552	6.96	**<0.001**
Glycemia	0.561	0.064	0.435–0.687	0.545	0.54.8	473	0.334
Corrected sodium *	0.600	0.061	0.481–0.719	0.585	0.533	145.3	0.118
Osmolality	0.648	0.058	0.534–0.761	0.635	0.500	306.7	**0.025**
BUN	0.664	0.056	0.553–0.774	0.645	0.48.3	21.6	**0.012**

Abbreviations: AUC—area under the curve; SE—standard error; 95%CI—95% confidence I=interval; NLR—neutrophil-to-lymphocyte ratio; BUN—blood urea nitrogen; * corrected for blood glucose. Statistically significant differences, with a probability value of *p* < 0.05, are represented in bold.

## Data Availability

Data can be made available upon reasonable request due to ethical restrictions.
